# Electrophysiological Assessment of Ulnar Nerve Function After Proximal and Distal Transulnar Coronary Angiography

**DOI:** 10.3390/jcm15135186

**Published:** 2026-07-02

**Authors:** Nimet Ucaroglu Can, Yusuf Can, Ibrahim Kocayigit, Emre Eynel, Ahmet Can Çakmak, Direnç Yılmaz, Mehmet Sirin Yıldız, Fahrettin Turna

**Affiliations:** 1 Department of Neurology, Sakarya Training and Research Hospital, Adapazari 54100, Turkey; 2 Department of Cardiology, Sakarya University, Adapazari 54100, Turkey; 3 Department of Cardiology, Sakarya Training and Research Hospital, Adapazari 54100, Turkeydrncylmzmd@gmail.com (D.Y.); fturna_53@hotmail.com (F.T.); 4 Cardiology Clinic, Pamukova State Hospital, Pamukova 54900, Turkey; 5 Cardiology Clinic, Tunceli State Hospital, Tunceli 62000, Turkey; mehmet_sirin65@hotmail.com

**Keywords:** transulnar access, coronary angiography, ulnar neuropathy, electrophysiology, nerve conduction study, distal ulnar access, peripheral nerve injury

## Abstract

**Background:** Transulnar access has emerged as a feasible alternative to transradial access for coronary angiography. However, the electrophysiological effects of proximal and distal transulnar approaches on ulnar nerve function remain insufficiently characterized. This study aimed to compare electrophysiological changes following proximal and distal transulnar coronary angiography and to evaluate their association with ulnar nerve involvement. **Methods:** This single-center retrospective observational study included 97 patients who underwent proximal (*n* = 56) or distal (*n* = 41) transulnar coronary angiography and developed post-procedural pain and/or paresthesia. Electroneuromyography and nerve conduction studies were performed before the procedure and at the fourth post-procedural week. Sensory and motor amplitudes, conduction velocities, and delta (Δ) changes were compared between groups. Generalized estimating equation analysis was additionally performed to evaluate longitudinal electrophysiological changes after adjustment for demographic and procedural variables. **Results:** No significant between-group differences were observed in pre- or post-procedural electrophysiological parameters. Although significant reductions in right ulnar sensory amplitude were observed over time, only 4 patients (4.1%) demonstrated post-procedural ulnar sensory amplitudes below the laboratory reference range, and no patient developed clinically significant ulnar neuropathy. Comparison of electrophysiological changes (Δ values) revealed no significant differences between proximal and distal transulnar access techniques. Generalized estimating equation analysis, adjusted for age and procedural characteristics, confirmed that the magnitude of electrophysiological changes over time was comparable between the two groups. **Conclusions:** Both proximal and distal transulnar access may be associated with mild, predominantly sensory, subclinical ulnar nerve involvement. However, distal transulnar access did not result in greater electrophysiological deterioration or additional neurological risk compared with proximal access.

## 1. Introduction

For many years, the femoral artery has been the primary access route for coronary angiography procedures. However, over the past two decades, upper-extremity arterial access routes have gained increasing importance. The growing preference for these approaches is largely attributable to improved patient comfort, lower procedural costs, and reduced length of hospital stay [1,2]. Nevertheless, transradial access is not entirely free of complications, with arterial spasm and anatomical variations representing important limitations [3,4]. Consequently, interest in alternative access routes has increased, bringing the transulnar approach into consideration.

Recent studies have increasingly emphasized the feasibility and safety of transulnar access in both coronary and neurovascular procedures. Transulnar access has been associated with lower rates of difficult vascular anatomy and may serve as an effective alternative when radial access is limited by vasospasm, tortuosity, or radial artery occlusion. Furthermore, a recent network meta-analysis demonstrated that upper-extremity access strategies, including ulnar access, were associated with lower bleeding and vascular complication rates compared with femoral access [5,6,7,8].

In recent years, the distal transulnar access technique has attracted increasing interest as an alternative vascular access method performed through the distal segment of the wrist. This approach is thought to offer several theoretical advantages, including reduced access-site complications, easier hemostasis, and preservation of the proximal arterial segment. Nevertheless, the more superficial course of the ulnar artery in the distal region and its close anatomical relationship with the ulnar nerve warrant careful consideration regarding the risk of neurovascular complications [9]. With the increasing number of endovascular procedures performed in recent years, peripheral nerve injuries associated with catheterization, particularly through upper-extremity arteries, have attracted growing attention. In procedures performed via the ulnar artery, neuropathy may develop through mechanisms such as compression, ischemia, hematoma formation, and direct mechanical trauma due to the close anatomical proximity of the ulnar nerve [10]. Although transulnar access has generally been associated with a low incidence of major complications, neurological symptoms such as transient paresthesia, sensory disturbances, and procedure-related peripheral nerve injury have been reported [11]. However, most available studies have relied primarily on clinical assessment, and objective electrophysiological evaluations of nerve function remain limited. 

Although clinical evaluation plays an important role in the diagnosis of ulnar neuropathy, electrophysiological examinations remain the cornerstone for confirming the diagnosis, localizing the lesion, and objectively assessing its severity. Nevertheless, the current literature evaluating neurological complications after vascular interventions relies predominantly on clinical findings, while objective electrophysiological comparisons remain limited. In particular, studies assessing post-procedural paresthesia and neuropathic symptoms using ENMG and nerve conduction studies are scarce [12,13]. 

In the present study, electrophysiological findings obtained before the procedure and four weeks after angiographic interventions performed via the ulnar artery were compared in patients who developed neurological symptoms following the procedure. In addition, the effects of proximal and distal access approaches on ulnar nerve function were evaluated. Through this approach, we aimed to objectively characterize intervention-related ulnar nerve involvement and provide stronger evidence regarding the safety of the transulnar approach.

## 2. Methods

### 2.1. Study Design and Patient Selection 

This single-center observational study was conducted through the retrospective analysis of patients who underwent proximal or distal transulnar coronary angiography at the Cardiology Clinic of Sakarya University Training and Research Hospital between June 2025 and May 2026.

Ethical approval for the study was obtained from the Ethics Committee of Sakarya University Faculty of Medicine (Decision No: 387, Date: 21 May 2026). The study was conducted in accordance with the principles of the Declaration of Helsinki. Because of the retrospective design of the study, the requirement for written informed consent was waived by the Ethics Committee.

Patients aged ≥18 years who underwent transulnar coronary angiography and reported pain and/or paresthesia on the ipsilateral side of the procedure were included in the study. Individuals with diabetes mellitus, malignancy, or a diagnosis of polyneuropathy were excluded. A total of 97 patients who met the inclusion criteria between June 2025 and May 2026 were enrolled. All patients underwent transulnar coronary angiography via the right ulnar artery using either the proximal or distal access approach. Because electrophysiological examinations were performed only in patients who developed post-procedural pain and/or paresthesia, the present study was designed to evaluate electrophysiological changes among symptomatic individuals rather than to determine the incidence of nerve involvement in the overall transulnar population. In our institution, ENMG examinations are not routinely performed in asymptomatic patients following transulnar procedures; therefore, asymptomatic individuals were not eligible for inclusion.

All included patients had previously undergone electrophysiological evaluation for various neurological complaints before coronary angiography, and only those with normal pre-procedural electrophysiological findings were eligible for inclusion. Following transulnar coronary angiography, patients who developed new-onset pain and/or paresthesia were referred for repeat electrophysiological assessment, which was performed at the fourth post-procedural week.

### 2.2. Transulnar Procedure

The right forearm was positioned in abduction and stabilized on a support attached to the catheterization laboratory table, while the wrist was maintained in hyperextension. To minimize the risk of injury related to the medial course of the ulnar nerve, subcutaneous local anesthesia with 1% lidocaine was administered lateral to the ulnar artery. Following arterial puncture using a 21-gauge, 5 cm needle, a 5F or 6F sheath was inserted over a 30–50 cm guidewire with a floppy tip and relatively rigid shaft. After sheath placement, 5000 IU of heparin and 100 μg of intra-arterial nitroglycerin were administered.

Upon completion of the procedure, arterial sheaths were removed immediately after diagnostic coronary angiography or percutaneous coronary intervention (PCI), and hemostasis was achieved using a Terumo (Tokyo, Japan) compression band system. Compression was maintained by inflating the band with 15–20 mL of air. Compression duration was approximately 2 h in the proximal transulnar access group and 1.5 h in the distal transulnar access group. The compression bands were subsequently removed, and patients without complications were discharged on the same day. Patients undergoing PCI were observed overnight and discharged in the absence of complications.

For diagnostic procedures, 5F Tiger catheters were used, whereas 6F Extra Backup, Judkins, or Amplatz guiding catheters were used during PCI procedures. All procedures were performed by a single experienced operator (Y.C.) with more than 10 years of experience, including over 5000 transradial and more than 1000 transulnar procedures. Because electrophysiological evaluations were performed four weeks after the procedure, the operator was not aware of the electrophysiological findings at the time of the intervention.

Patients who had undergone ulnar angiography within the preceding month and subsequently developed right arm pain and/or numbness were referred for electroneuromyography (ENMG) evaluation to assess possible nerve injury. Demographic data, including age and sex, were obtained from hospital records.

### 2.3. Electrophysiological Assessment 

Electrophysiological studies were performed using a Nihon Kohden (Tokyo, Japan) EMG system. All assessments were conducted at room temperature. Sensory nerve conduction studies were performed using the antidromic technique. Median sensory responses were recorded from the second digit, whereas ulnar sensory responses were recorded from the fifth digit. In motor conduction studies, median nerve responses were recorded from the abductor pollicis brevis muscle and ulnar nerve responses from the abductor digiti minimi muscle. Nerve stimulations were delivered using supramaximal stimulation. Sensory and motor amplitudes, latencies, and conduction velocities were evaluated. The results were interpreted according to our laboratory reference values. All electrophysiological assessments were performed by an experienced neurologist who was blinded to the vascular access site (proximal or distal transulnar access).

**Figure 1:**
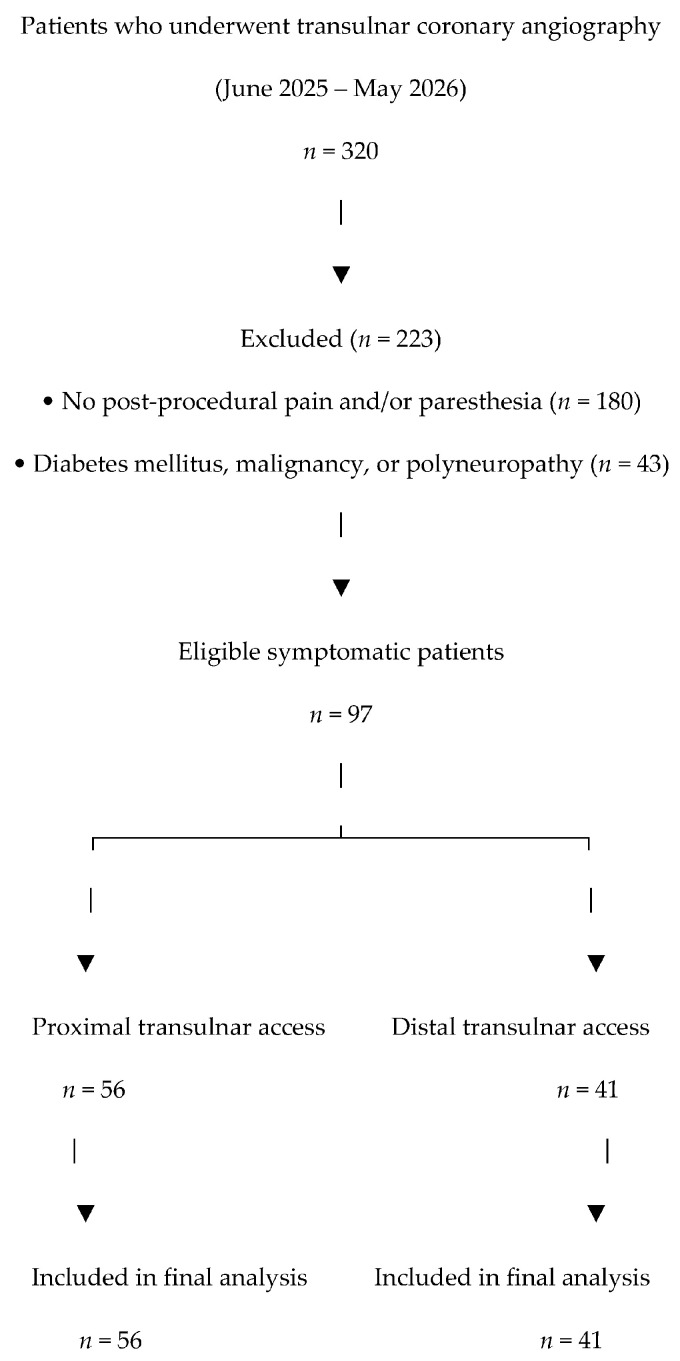
Flow diagram of patient selection and study enrollment.

Subsequently, nerve conduction parameters obtained before the procedure and at the fourth week following the procedure were compared between patients who underwent proximal and distal transulnar angiography (Figure 1).

### 2.4. Statistical Analysis

All statistical analyses were performed using IBM SPSS Statistics version 28.0 (IBM Corp., Armonk, NY, USA). The distribution of continuous variables was assessed using the Kolmogorov–Smirnov and Shapiro–Wilk tests. Normally distributed variables are presented as mean ± standard deviation (SD), whereas non-normally distributed variables are expressed as median (minimum–maximum). Categorical variables are presented as frequencies and percentages.

The study population was divided into two groups according to the vascular access site: the proximal transulnar access group (Group 1) and the distal transulnar access group (Group 2).

For between-group comparisons, the independent samples *t*-test was used for normally distributed continuous variables, whereas the Mann–Whitney *U* test was applied for non-normally distributed variables. Categorical variables were compared using Pearson’s chi-square test or Fisher’s exact test, as appropriate.

- To evaluate procedure-related electrophysiological changes, delta (Δ) values were calculated for each electrophysiological parameter according to the following formula:
Δ value = Post-procedural value − Pre-procedural value

Between-group comparisons of Δ values were performed using the independent samples *t*-test or Mann–Whitney *U* test according to data distribution.

To further evaluate longitudinal electrophysiological changes while accounting for repeated measurements, generalized estimating equation (GEE) models were constructed with group (proximal vs. distal), time (pre- vs. post-procedural), and the group × time interaction as fixed effects. The models were adjusted for age, puncture attempts, puncture duration, sheath size, and procedure duration. Group effects represented overall differences between proximal and distal transulnar access groups, time effects represented overall pre- to post-procedural changes, and group × time interaction effects represented differential electrophysiological changes over time between the two access techniques.

An a priori power analysis was performed using G*Power version 3.1.9.7. Based on an effect size (dz = 0.467) derived from a comparable electrophysiological study evaluating ulnar sensory amplitude changes, a minimum sample size of 65 participants was required to achieve 95% statistical power with a two-sided α level of 0.05 using the Wilcoxon signed-rank test. The final study population consisted of 97 patients, exceeding the calculated sample size requirement and corresponding to an estimated statistical power of 95.1%.

All statistical tests were two-sided, and a *p* value <0.05 was considered statistically significant.

## 3. Results

A total of 97 patients were included in the study, comprising 56 patients in the proximal transulnar access group and 41 patients in the distal transulnar access group. No significant differences were observed between the groups regarding age, sex, diabetes mellitus, smoking status, antiplatelet therapy, anticoagulant therapy, or sheath size (all *p* > 0.05). However, hypertension and hyperlipidemia were more prevalent in the proximal access group (*p* = 0.010 and *p* = 0.007, respectively). Procedural characteristics differed between the groups, with the distal transulnar access group demonstrating a higher number of puncture attempts, longer puncture duration, and longer procedure duration (*p* = 0.029, *p* = 0.037, and *p* = 0.022, respectively). No access-site hematoma was observed in either group. Baseline demographic, clinical, and procedural characteristics are summarized in Table 1.

Pre-procedural electrophysiological findings were generally comparable between the proximal and distal transulnar access groups. No statistically significant between-group differences were observed in baseline sensory or motor nerve conduction parameters. 

Baseline electrophysiological characteristics of the study groups are presented in Table 2.

Post-procedural electrophysiological findings are presented in Table 3. Overall, sensory and motor nerve conduction parameters were comparable between the proximal and distal transulnar access groups. No significant between-group differences were observed for median nerve conduction measurements or most ulnar nerve parameters. However, right ulnar motor amplitude was lower in the distal transulnar access group compared with the proximal group. However, this isolated between-group difference was not supported by the delta analysis or the generalized estimating equation analysis, both of which demonstrated comparable electrophysiological changes over time between the two access techniques. 

Detailed post-procedural electrophysiological findings are summarized in Table 3.

To evaluate procedure-related electrophysiological changes, differences between post-procedural and baseline measurements (Δ values) were compared between the proximal and distal transulnar access groups. No significant between-group differences were observed in the magnitude of change for any sensory or motor nerve conduction parameter, including median and ulnar nerve amplitudes and conduction velocities (all *p* > 0.05). 

Detailed results of the comparison of electrophysiological changes (Δ values) between the proximal and distal transulnar access groups are presented in Table 4.

Generalized estimating equation analysis was performed to evaluate the effects of access site, time, and their interaction on electrophysiological parameters after adjustment for age, puncture attempts, puncture duration, sheath size, and procedure duration. Significant time effects were observed for right ulnar sensory amplitude (*p* < 0.001), right ulnar sensory conduction velocity (*p* = 0.048), right median motor conduction velocity (*p* = 0.011), right ulnar motor amplitude (*p* = 0.014), left ulnar sensory amplitude (*p* = 0.007), and left ulnar motor amplitude (*p* = 0.034). However, no significant group × time interactions were identified for any electrophysiological parameter. 

Detailed results of the generalized estimating equation analysis are presented in Table 5.

**Figure 2:**
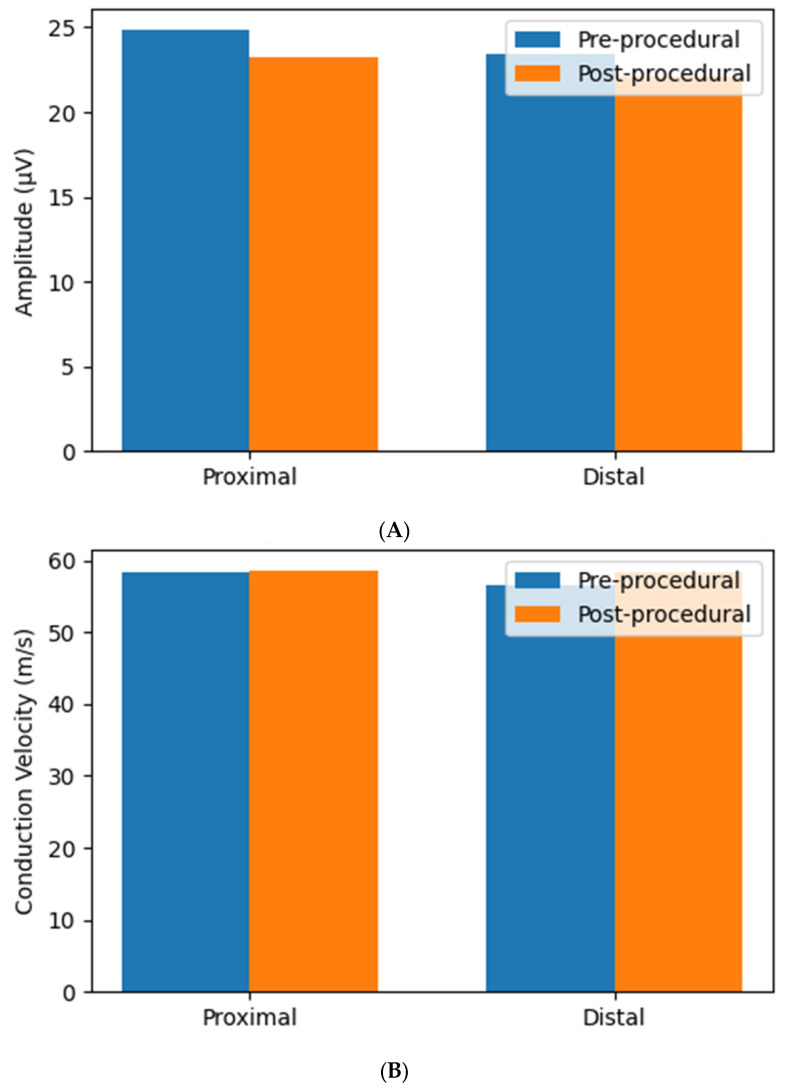
(**A**) Comparison of pre- and post-procedural right ulnar sensory amplitudes in proximal and distal transulnar access groups. (**B**) Comparison of pre- and post-procedural right ulnar sensory conduction velocities in proximal and distal transulnar access groups.

The changes in right ulnar sensory amplitudes and sensory conduction velocities before and after the procedure are illustrated in Figure 2A and Figure 2B, respectively.

## 4. Discussion

In this study, electrophysiological changes were evaluated in patients who developed pain and/or paresthesia following proximal or distal transulnar coronary angiography. The principal finding of our study was that although a significant reduction in right ulnar sensory amplitude was observed following both access techniques, the distal transulnar approach did not confer additional neurological risk compared with proximal access. Furthermore, both the delta analyses and the generalized estimating equation analyses consistently demonstrated that distal transulnar access was not associated with greater electrophysiological deterioration than proximal transulnar access. Although minor changes were observed in certain median motor conduction parameters, the magnitude of these alterations was limited and was not supported by between-group delta analyses.

The safety and feasibility of transulnar access have been demonstrated in several previous studies. Roghani-Dehkordi et al. reported no significant differences in complication rates between transulnar and transradial access [14]. Similarly, in a meta-analysis conducted by Sedhom et al., transulnar access was shown to be non-inferior to transradial access with respect to procedural success and complication rates [15]. In the AJULAR studies conducted by Gokhroo et al., transulnar access was also reported to be associated with high technical success rates and low rates of vascular complications [16,17]. The absence of major neurological complications and the finding that distal transulnar access was not associated with greater electrophysiological deterioration are consistent with the safety data reported in the existing literature.

The development of neurological complications in transulnar access is anatomically related to the close proximity between the ulnar artery and the ulnar nerve. From an anatomical perspective, the ulnar nerve accompanies the ulnar artery along much of the forearm and wrist, resulting in close neurovascular proximity at the puncture site. Consequently, local tissue edema, perivascular inflammation, vascular spasm, or small hematomas may affect adjacent neural structures even in the absence of direct nerve trauma. This anatomical relationship may explain why sensory fibers appear more vulnerable than motor fibers and may contribute to the mild reductions in sensory amplitudes observed after both proximal and distal transulnar access procedures. Potential mechanisms include direct mechanical trauma during puncture, local hematoma formation, compression, transient ischemia, and perineural inflammation [10]. In our study, the significant reduction in right ulnar sensory amplitude observed in both groups suggests that sensory fibers may be more susceptible to procedure-related mechanical and ischemic injury. In contrast, the absence of substantial impairment in motor conduction parameters supports the notion that the neurological involvement was predominantly mild and transient in nature. The relative preservation of motor fibers suggests a mechanism of mild transient neuropraxia rather than severe axonal injury or advanced conduction block. Although statistically significant reductions in ulnar sensory amplitudes were observed, the magnitude of these changes was relatively small and predominantly electrophysiological in nature. No patient developed clinically overt motor deficit or severe neurological impairment requiring additional intervention. Furthermore, most patients reported substantial improvement or resolution of paresthesia by the fourth post-procedural week. Therefore, the observed findings are more consistent with mild subclinical sensory nerve involvement than with clinically significant ulnar neuropathy. These findings suggest that the observed electrophysiological alterations are unlikely to have substantial clinical consequences in most patients. 

Although a direct causal relationship between post-procedural pain or paresthesia and the observed electrophysiological abnormalities cannot be definitively established, the reduction in ulnar sensory amplitudes among symptomatic patients suggests a possible association between sensory complaints and mild ulnar nerve involvement. However, because symptom severity was not quantitatively assessed and no asymptomatic control group was available, these findings should be interpreted with caution.

Todnem et al. reported that extended electrophysiological examinations have high sensitivity for detecting mild ulnar neuropathies [18]. Similarly, Jang et al. identified electrophysiological alterations in the superficial radial nerve following transradial catheterization that were not clinically apparent [13]. Consistent with these findings, the demonstration of amplitude reduction accompanying clinical symptoms in our study further supports the role of electrophysiological assessment as an important tool for detecting subclinical peripheral nerve injury following interventional procedures. Previous studies evaluating transradial access have also reported neurological complications involving adjacent peripheral nerves. Although such complications are generally uncommon, transient sensory disturbances, numbness, and electrophysiological abnormalities have been described following radial artery catheterization. Similar to the findings reported after transradial procedures, the predominantly sensory and subclinical nature of the electrophysiological alterations observed in our study suggests that minor nerve irritation rather than severe structural nerve injury may occur following upper-extremity arterial access procedures. These observations support the concept that peripheral nerve involvement associated with vascular access is often mild, transient, and detectable primarily through electrophysiological assessment [2].

One of the important findings of our study is that distal transulnar access did not confer additional neurological risk compared with proximal access. Distal access theoretically offers several advantages, including a more superficial anatomical location, shorter hemostasis duration, and preservation of the proximal arterial segment. Nevertheless, concerns have been raised regarding a potentially increased risk of neurological complications due to the closer anatomical relationship between the distal ulnar artery segment and the ulnar nerve. Manzoor et al. reported that distal ulnar access is feasible for neurointerventional procedures [19]. Our findings further demonstrate that distal access has a safety profile comparable to that of proximal access from an electrophysiological perspective. Moreover, both the delta analyses and generalized estimating equation analyses consistently demonstrated that distal transulnar access was not associated with greater electrophysiological deterioration than proximal transulnar access, further supporting the neurological safety of the distal approach.

Dossani et al. demonstrated that ulnar artery access is safe and feasible for neurointerventional procedures [20]. Similarly, de Andrade et al. reported that transulnar access can be used in coronary interventions with low complication rates [21]. Our study supports these findings from a neurological perspective and contributes to the existing literature, particularly through the use of objective electrophysiological assessment. While neurological complications following transulnar interventions have largely been reported on the basis of clinical evaluation alone, data comparing pre- and post-procedural nerve conduction studies remain limited. Therefore, our study may represent one of the few investigations directly comparing the neurological effects of distal and proximal transulnar access using objective electrophysiological methods.

Previous studies evaluating transulnar access in neurovascular and coronary procedures similarly reported high procedural success rates, with low incidence of major vascular or neurological complications, supporting the overall safety profile of transulnar access [6,22].

From a clinical perspective, our findings suggest that both proximal and distal transulnar access techniques can be performed with a low risk of clinically significant neurological complications. In patients who develop post-procedural paresthesia or numbness, electrophysiological assessment may be useful for identifying mild sensory nerve involvement and distinguishing transient nerve irritation from clinically significant ulnar neuropathy. Furthermore, the finding that distal transulnar access was not associated with additional neurological risk may support its continued use as an alternative vascular access strategy when clinically indicated. These findings may assist clinicians in patient counseling, post-procedural follow-up, and the management of neurological complaints following transulnar interventions.

This study has several limitations. First, it was a single-center retrospective observational study with a relatively limited sample size. Therefore, the possibility of unmeasured confounding and information bias cannot be completely excluded. Second, an important limitation is the inclusion of only symptomatic patients who reported post-procedural pain and/or paresthesia. Consequently, selection bias cannot be excluded, and the findings may not be generalizable to all patients undergoing transulnar coronary angiography. Furthermore, because asymptomatic individuals were not evaluated electrophysiologically, the true incidence of subclinical nerve involvement within the overall transulnar access population could not be determined. Another important limitation is the absence of an asymptomatic control group. Therefore, direct comparisons between symptomatic and asymptomatic patients regarding electrophysiological changes could not be performed. In addition, symptom severity was not quantitatively assessed using the Visual Analog Scale (VAS) or validated neuropathic pain scales. Finally, the relatively short follow-up period precluded the evaluation of long-term neurological outcomes.

## 5. Conclusions

In conclusion, this study demonstrated that both distal and proximal transulnar access may be associated with mild, predominantly sensory, subclinical ulnar nerve involvement, while distal access did not confer additional neurological risk compared with proximal access. Electrophysiological assessment may serve as an important tool for the objective detection of mild or subclinical peripheral nerve injury following interventional procedures. To the best of our knowledge, this study is among the first to compare distal and proximal transulnar access using pre- and post-procedural electrophysiological evaluation.

## Figures and Tables

**Table 1:** Baseline demographic, clinical, and procedural characteristics according to access site.

Variable	Proximal (*n* = 56)	Distal (*n* = 41)	*p* Value
Age, years	55 (38–67)	56 (40–66)	0.612
Male sex, *n* (%)	42 (75.0)	32 (78.1)	0.915
Hypertension, *n* (%)	46 (82.1)	23 (56.1)	0.010
Diabetes mellitus, *n* (%)	19 (33.9)	8 (19.5)	0.182
Hyperlipidemia, *n* (%)	20 (35.7)	4 (9.8)	0.007
Previous stroke, *n* (%)	5 (8.9)	0 (0.0)	0.071
Smoking, *n* (%)	26 (46.4)	18 (43.9)	0.968
Antiplatelet therapy, *n* (%)	23 (41.1)	12 (29.3)	0.326
Anticoagulant therapy, *n* (%)	6 (10.7)	1 (2.4)	0.233
Sheath size, *n* (%)			0.185
5F	13 (23.2)	16 (39.0)	
6F	43 (76.8)	25 (61.0)	
Number of puncture attempts	1 (1–5)	2 (1–4)	0.029
Puncture duration, sec	20 (10–120)	30 (15–60)	0.037
Procedure duration, min	8 (2–41.6)	10 (6–45)	0.022

Values are presented as median (minimum–maximum) or *n* (%), as appropriate.

**Table 2:** Pre-procedural electrophysiological findings according to access site.

Parameter	Proximal Transulnar (*n* = 56)	Distal Transulnar (*n* = 41)	*p* Value
Right median sensory amplitude	26.94 ± 8.37	24.76 ± 5.67	0.321
Right median sensory conduction velocity	56.69 ± 4.07	57.97 ± 3.99	0.126
Right ulnar sensory amplitude	24.82 ± 7.23	23.42 ± 6.58	0.333
Right ulnar sensory conduction velocity	58.21 ± 4.41	56.47 ± 4.00	0.050
Right median motor amplitude	9.14 ± 1.94	9.17 ± 1.78	0.729
Right median motor conduction velocity	55.20 ± 3.38	55.09 ± 3.26	0.939
Right ulnar motor amplitude	8.98 ± 1.73	8.39 ± 1.49	0.087
Right ulnar motor conduction velocity	57.19 ± 4.51	58.49 ± 3.95	0.101
Left median sensory amplitude	28.03 ± 7.17	26.07 ± 6.44	0.167
Left median sensory conduction velocity	56.46 ± 4.15	57.68 ± 3.65	0.135
Left ulnar sensory amplitude	24.83 ± 6.59	24.35 ± 6.10	0.881
Left ulnar sensory conduction velocity	57.25 ± 4.26	57.76 ± 3.93	0.546
Left median motor amplitude	9.09 ± 1.53	9.13 ± 1.83	0.890
Left median motor conduction velocity	55.99 ± 3.75	56.23 ± 3.66	0.659
Left ulnar motor amplitude	9.14 ± 1.60	8.72 ± 1.63	0.153
Left ulnar motor conduction velocity	58.36 ± 4.09	57.87 ± 4.02	0.470

Values are presented as mean ± standard deviation.

**Table 3:** Post-procedural electrophysiological findings according to access site.

Parameter	Proximal Transulnar (*n* = 56)	Distal Transulnar (*n* = 41)	*p* Value
Right median sensory amplitude	26.90 ± 8.49	24.68 ± 5.59	0.168
Right median sensory conduction velocity	56.96 ± 4.37	57.68 ± 4.91	0.132
Right ulnar sensory amplitude	23.20 ± 7.09	22.09 ± 6.41	0.324
Right ulnar sensory conduction velocity	58.46 ± 5.41	58.29 ± 4.33	0.134
Right median motor amplitude	9.14 ± 2.05	9.09 ± 1.91	0.867
Right median motor conduction velocity	56.62 ± 4.38	56.29 ± 4.49	0.937
Right ulnar motor amplitude	8.85 ± 1.80	8.21 ± 1.57	0.027
Right ulnar motor conduction velocity	57.49 ± 4.11	58.02 ± 3.69	0.257
Left median sensory amplitude	27.95 ± 7.21	26.03 ± 6.19	0.233
Left median sensory conduction velocity	56.47 ± 4.00	57.63 ± 5.12	0.137
Left ulnar sensory amplitude	24.46 ± 6.71	23.80 ± 6.36	0.652
Left ulnar sensory conduction velocity	57.20 ± 4.07	58.33 ± 3.85	0.615
Left median motor amplitude	9.11 ± 1.58	9.10 ± 1.92	0.889
Left median motor conduction velocity	56.03 ± 3.82	56.36 ± 4.42	0.728
Left ulnar motor amplitude	9.01 ± 1.72	8.64 ± 1.67	0.173
Left ulnar motor conduction velocity	57.87 ± 4.79	59.11 ± 4.58	0.529

Values are presented as mean ± standard deviation.

**Table 4:** Comparison of electrophysiological changes (Δ values) between proximal and distal transulnar access groups.

Parameter	Proximal Transulnar (Δ)	Distal Transulnar (Δ)	*p* Value
Right median sensory amplitude	0 (−9.8 to 6.1)	0 (−3.2 to 5.8)	0.602
Right median sensory conduction velocity	−0.26 ± 4.92	0.30 ± 4.76	0.575
Right ulnar sensory amplitude	0 (−6.5 to 13.4)	0.3 (−6.0 to 10.0)	0.504
Right ulnar sensory conduction velocity	−0.25 ± 5.24	−1.82 ± 5.08	0.144
Right median motor amplitude	0 (−1.7 to 0.5)	0 (−1.2 to 1.7)	0.635
Right median motor conduction velocity	−1.25 (−13.2 to 11.1)	0 (−14.1 to 6.3)	0.463
Right ulnar motor amplitude	0 (−0.6 to 2.6)	0 (−1.0 to 4.0)	0.821
Right ulnar motor conduction velocity	−0.30 ± 4.99	0.46 ± 4.84	0.451
Left median sensory amplitude	0 (−8.6 to 5.3)	0 (−5.4 to 2.8)	0.499
Left median sensory conduction velocity	−0.01 ± 5.41	0.05 ± 4.98	0.953
Left ulnar sensory amplitude	0 (−2.7 to 8.4)	0 (−1.7 to 8.2)	0.852
Left ulnar sensory conduction velocity	0.05 ± 4.77	−0.57 ± 4.32	0.517
Left median motor amplitude	0 (−1.3 to 1.5)	0 (−0.5 to 1.2)	0.677
Left median motor conduction velocity	−0.04 ± 4.54	−0.13 ± 4.80	0.927
Left ulnar motor amplitude	0 (−0.5 to 1.7)	0 (−0.7 to 2.9)	0.275
Left ulnar motor conduction velocity	0.49 ± 5.33	−1.24 ± 5.90	0.133

Δ values were calculated as post-procedural minus pre-procedural measurements. Data are presented as mean ± standard deviation or median (minimum–maximum), as appropriate.

**Table 5:** Generalized estimating equation analysis of electrophysiological parameters according to group and time effects.

Parameter	Group *p*	Time *p*	Group × Time *p*
Right median sensory amplitude	0.168	0.698	0.892
Right median sensory CV	0.132	0.973	0.567
Right ulnar sensory amplitude	0.324	<0.001	0.655
Right ulnar sensory CV	0.134	0.048	0.135
Right median motor amplitude	0.867	0.289	0.289
Right median motor CV	0.937	0.011	0.826
Right ulnar motor amplitude	0.027	0.014	0.656
Right ulnar motor CV	0.257	0.871	0.442
Left median sensory amplitude	0.233	0.700	0.870
Left median sensory CV	0.137	0.974	0.952
Left ulnar sensory amplitude	0.652	0.007	0.610
Left ulnar sensory CV	0.615	0.573	0.504
Left median motor amplitude	0.889	0.832	0.488
Left median motor CV	0.728	0.862	0.927
Left ulnar motor amplitude	0.173	0.034	0.712
Left ulnar motor CV	0.529	0.515	0.132

Generalized estimating equation models were adjusted for age, puncture attempts, puncture duration, sheath size, and procedure duration. Group *p* values represent overall differences between proximal and distal access groups. Time *p* values represent overall pre- to post-procedural changes. Group × Time *p* values represent differential changes between groups.

## Data Availability

The raw data supporting the conclusions of this article will be made available by the authors on request.
